# Suppressing maize stalk rot through promoted rhizosphere microbial recruitment in cultivar mixtures

**DOI:** 10.3389/fmicb.2025.1627153

**Published:** 2025-12-02

**Authors:** Panpan An, Xucun Jia, Moubiao Zhang, Kui Liu, Xiuling Wang, Hongping Li, Yali Zhao, Qun Wang, Mimi Xie, Tianxue Liu, Chaohai Li

**Affiliations:** 1 Agronomy College, Collaborative Innovation Center of Henan Grain Crops/Co-construction State Key Laboratory of Wheat and Maize Crop Science, Henan Agricultural University, Zhengzhou, Henan, China; 2 Swift Current Research and Development Centre, Swift Current, SK, Canada; 3 Henan Province Agro-ecosystem Field Observation and Research Station, Xiping, China

**Keywords:** cultivar mixtures, stalk rot, microbial community, co-occurrence network, keystone taxa

## Abstract

**Introduction:**

Crop diversification is a critical strategy for improving resource use efficiency and suppressing disease. Mixing cultivars from the same crop is one approach to diversifying planting patterns. However, it remains unknown how mixing cultivars in maize affects the soil microbial community, stalk rot incidence, and grain yield.

**Methods:**

A 2-year field experiment was performed, which included one stalk rot-resistance cultivar, DH662; one stalk rot-susceptible cultivar, DH701; and a mixture of DH662 and DH701.

**Results:**

Cultivar mixtures and DH662 monocrop had lower disease incidence and higher grain yield than DH701 monocrop. The microbial community structure in both rhizosphere and bulk soil was notably impacted by the resistant cultivar as well as the cultivar mixtures. Distinct modules within the rhizosphere microbial community co-occurrence network were identified, differentiating cultivar mixtures from DH701 monocrops. The network structure of the rhizosphere in cultivar mixtures closely resembled that observed in DH662 monocrops. Keystone taxa were higher in cultivar mixtures compared to their abundance in DH701 monocrops. The keystone taxa (*Adhaeribacter* and *Gemmatimonas*) were positively related to grain yield and negatively correlated with disease incidence.

**Discussion:**

Overall, our results demonstrated that cultivar mixtures had a substantial impact on the assembly and keystone taxa of microbial communities in the rhizosphere and bulk soil, leading to a reduction in stalk rot occurrence and an increase in grain yield. This study demonstrated the potential for maize yield improvement through cultivar mixtures. These findings improved insights into how beneficial microbial communities in the rhizosphere contributed to the positive effects observed in cultivar mixtures.

## 1 Introduction

Maize is a fundamental grain crop and holds growing importance in ensuring food security worldwide. Globally, stalk rot is a universal soil-borne fungal disease with destructive potential for maize (Yu et al., 2017), leading to grain yield loss and quality deterioration. Maize stalk rot was first identified in China during the 1920s and has since become a significant threat to maize production (Yang et al., 2010). It has caused a yield losses of approximately 5% per year. In certain circumstances, yield loss caused by stalk rot diseases has increased by 10–20% or even to no yield (Yu et al., 2017). Apart from yield loss, stalk rot weakened the stem, making it prone to lodging and difficult to harvest (Freije and Wise, 2016).

Crop diversification with greater genetic and/or functional diversity could achieve production stability by enhancing crop protection and improving productivity using various systems such as intercropping and cultivar mixtures (Hufnagel et al., 2020). Intercropping or cultivar mixtures are important ways to facilitate sustainable agricultural production, which involves cultivating two or more crops in the same space simultaneously (Lithourgidis et al., 2011). In general, cultivar mixtures or intercropping have been widely adopted for thousands of years by small-scale farmers in Latin America, Africa, and Asia, and they could improve aboveground productivity and then increase grain yield, due to spatial eco-niches complementary (Cong et al., 2015; Gou et al., 2016; Zhang et al., 2020).

Crop monocrop is more vulnerable to diseases than intercropping due to the accumulation of host-specific pathogens. Intercropping reduced disease incidence by 55%, compared with monocrops (Chadfield et al., 2022). The increased plant diversity of intercropping promotes crop soil-borne disease resistance through the direct inhibitory action and the dilution effect (Mommer et al., 2018; Wang et al., 2023; van Ruijven et al., 2020; Ampt et al., 2022). The assembly and function of microbial communities in the rhizosphere are strongly connected to sustaining crop health. Plants under biotic stress actively recruit beneficial microbes to the rhizosphere to prevent the spread of soil pathogens (Rolfe et al., 2019; Carrión et al., 2019). Plant-beneficial microbes tend to be more diverse and abundant in diversified planting patterns. Plants could change their rhizosphere-dominant microbes by perceiving neighboring plants’ root exudation (Li et al., 2016; Ulbrich et al., 2022). When intercropped with potato onion, tomato improves rhizosphere microbiome recruitment and establishes a disease-suppressive microbial community through potato onion root exudates (Zhou et al., 2023). Intercropping can be competition (Lowry et al., 2020), or facilitation (Kong et al., 2022) and also affects plant nutrient uptake by modulating soil microbiome diversity and community composition (Jiang et al., 2024). To a certain degree, the competition for resources, e.g., light and nutrients, in intercropping induced plant immune disease resistance (Zhu and Morel, 2019).

Intraspecific intercropping or cultivar mixtures also present a promising opportunity for sustainable and intensive agriculture. A few studies have shown that mixing different varieties or genotypes of the same crop species can utilize genetic diversity to enhance intraspecific diversity advantages. In terms of abiotic stress, cultivar mixtures between lodging-resistant and lodging-susceptible wheat cultivars could reduce to some extent the occurrence of lodging as a consequence of the support of the lateral and longitudinal derived from lodging-resistant cultivar (Cai et al., 2019). Cultivar mixtures of different maize hybrids took advantage of the border effect and increased grain weight (Wang et al., 2017). Cultivar mixtures could enhance ecosystem functioning under rainfed and lower soil fertility conditions, with lower productivity, partly through complementarity effects (Su et al., 2023). With regard to biotic stress, different genotypes had different disease resistance (Finckh et al., 2000; Zhu et al., 2000; Ye et al., 2013; Ye et al., 2019) and resource utilization efficiency (Neugschwandtner and Kaul, 2015; Kamran et al., 2018), which can ecosystem functioning can be enriched with cultivar mixtures and enhance ecosystem productivity and stability. Intraspecific crop diversification provided an ecological strategy to control diseases, which can significantly contribute to the sustainability of crop production (Zhu et al., 2000). Therefore, intercropping and mixed cropping appear to be a promising approach to combat soil-borne diseases. Cultivar mixtures comprising different maize varieties can elevate grain yield stability and facilitation, particularly under the challenges posed by climate change. The processes and functions governing the formation of a rhizosphere microbial community were likely closely linked to sustaining plant health. However, we still lack an understanding of the assembly mechanisms of these microorganisms and how their functions change during the deterioration of soil-borne diseases.

To address the issue, we investigated the characteristics of rhizosphere and soil microbiome communities associated with Fusarium wilt disease (caused by Fusarium species). We evaluated their assembly under different planting patterns. We hypothesized that (1) in comparison with monocrop, the grain yield of maize would be improved in cultivar mixtures, (2) cultivar mixtures induce changes in the rhizosphere but not bulk soil microbial communities by improving microbial diversity, and (3) cultivar mixtures promoted the recruitment of rhizosphere healthy communities of susceptible varieties through keystone taxa and enhanced disease resistance.

## 2 Materials and methods

### 2.1 Site description

The field experiments were carried out at Henan Agricultural University Research Farm in Zhengzhou, Henan Province, China (34°48′N, 113°36′E), with an annual precipitation of 609.5 mm, average annual temperature of 14.7 °C, and a frost-free period of 216 days. Maize was grown under irrigated conditions.

The field experiments were carried out from June to October in 2022 and 2023. The soil was classified as tidy soil, with an initial status in Table 1. Basal fertilization of urea (90 kg N ha^−1^), calcium superphosphate (120 kg P ha^−1^), and potassium sulfate (50 kg N ha^−1^) was incorporated at a 10 cm depth at sowing. Urea topdressing at a rate of 135 kg N ha^−1^ was applied at the V10 leaf stage.

**Table 1:** Initial status of the soil before planting.

Soil organic matter (g kg^−1^)	Total nitrogen (g kg^−1^)	Available nitrogen (mg kg^-1^)	Available phosphorus (mg kg^-1^)	Available potassium (mg kg^-1^)
17.3	1.01	74.3	17.1	217.0

### 2.2 Experimental design

**Figure 1:**
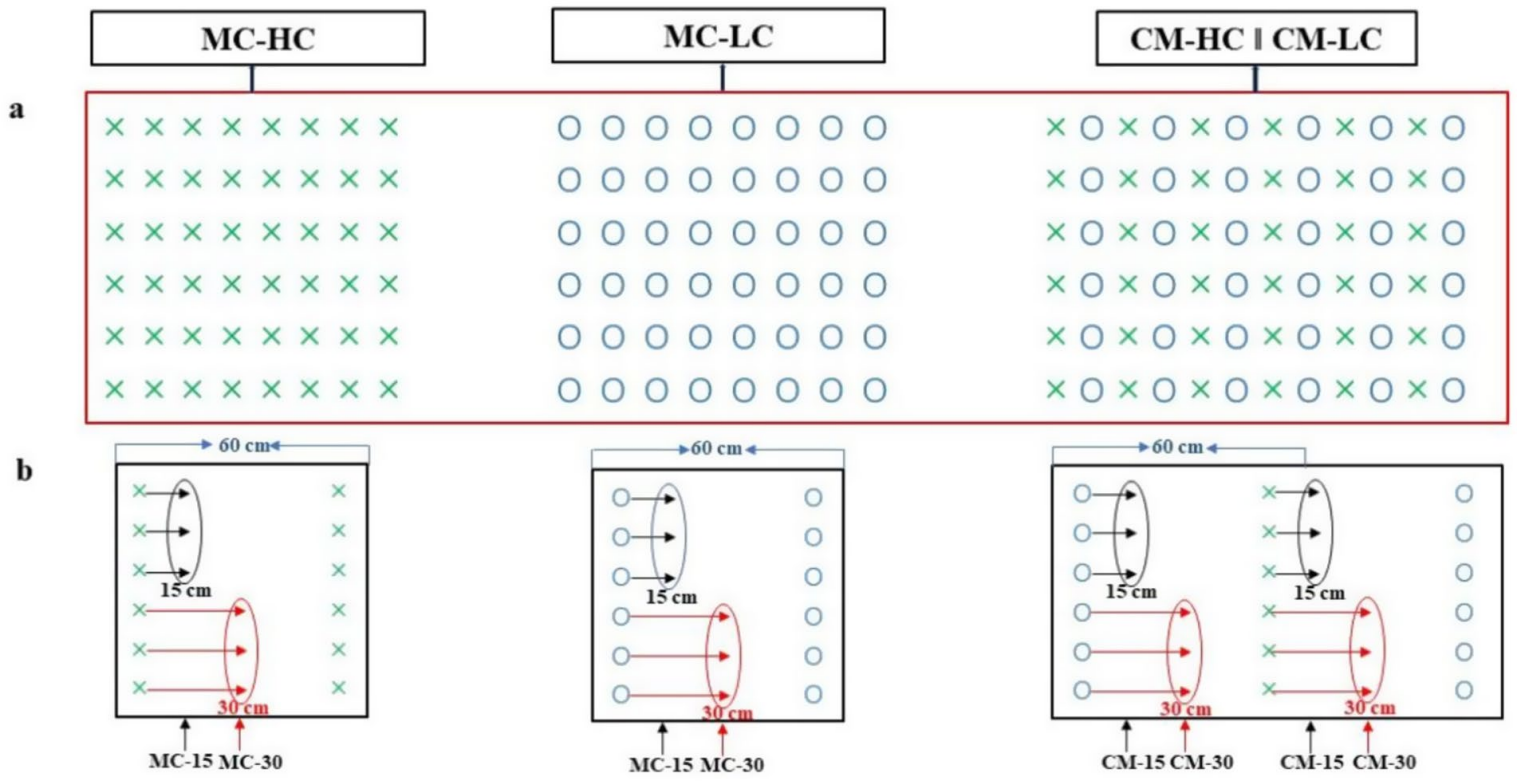
Schematic diagram of the field layout illustrates various planting patterns **(a)** and a schematic diagram of the bulk soil sampling under these planting patterns **(b)**. ×, HC, DH662-the resistant variety; ○, LC, DH701-the susceptible variety; MC, monocrop; CM, cultivar mixtures; MC-HC, DH662 under monocrop; MC-LC, DH701 under monocrop; CM-HC, DH662 under cultivar mixtures; CM-LC, DH701 under cultivar mixtures. MC-15, the soil collected 15 cm away from the roots under monocrop; MC-30, the soil collected 30 cm away from the roots under monocrop; CM-15, the soil collected 15 cm away from the roots under cultivar mixtures; CM-30, the soil collected 30 cm away from the roots under cultivar mixtures.

Two widely cultivated maize hybrids, DengHai662 (DH662) and DengHai701 (DH701), were selected for this study. Both cultivars have similar maturation times, with DH662 being resistant to stalk rot (HC) and DH701 being susceptible (LC). The study involved two planting patterns—monocropping and cultivar mixtures—resulting in four experimental treatments: monocrop DH662 (MC-HC), monocrop DH701 (MC-LC), cultivar mixture of DH662 and DH701 (CM-HC), and cultivar mixture of DH701 and DH662 (CM-LC) (Figure 1).

To ensure uniform plant population density, all treatments were thinned at the V4 leaf stage to maintain a consistent density of 75,000 plants per hectare. The experiment employed a fully randomized design, including three repetitions for each treatment. Each plot measured 48 m^2^ (8 m × 6 m) and consisted of 10 rows, with a spacing of 0.60 m between rows. Other field management practices were consistent with those typically employed in high-yield agricultural systems.

### 2.3 Artificial inoculation and symptom scoring in the field

The selected high-quality maize kernels were sterilized in an autoclave and inoculated with *F. graminearum* (provided by Prof. Junjie Hao, Henan Academy of Agricultural Sciences), and then incubated at 25 °C in complete darkness under a constant temperature incubator for 2 weeks. Artificial inoculation was carried out at the V12 leaf stage. Before inoculation, the maize kernels, which were coated with mycelium, were collected and mixed thoroughly to ensure a consistent inoculation process. To carry out the artificial inoculation effectively, 50 to 70 g of the infected maize kernels were buried in a hole situated 5 to 10 cm from the base of each maize plant. Then it was irrigated in order to raise soil humidity to facilitate fungal mycelium growth and infection (Mims and Vaillancourt, 2002; Yang et al., 2004).

### 2.4 Incidence of disease

The maize plants were evaluated for symptoms of stalk rot three times at weekly intervals, beginning 4 weeks after inoculation. The following distinctive features of stalk rot were recorded: browning in the lower internodes, spongy stem texture, wilting, ear dropping, stem lodging, and plant death (Yang et al., 2010; Ye et al., 2019). The incidence of stalk rot disease was calculated using the following formula:


$$\mathrm{ID}\;(\text{incidence of disease})=\frac{n}{N}\times 100\%$$

*N* is the total number of plants, and *n* is the number of diseased plants.

### 2.5 Plant sampling and analysis

For all plots, two rows with a length of 8 m were harvested for grain yield at maturity every year. Before harvest, we measured shoot biomass by sampling five plants from each plot. The shoot biomass was dried at 75 °C for 72 h and weighed.

### 2.6 Soil sampling and analysis

Rhizosphere and bulk soils from healthy and diseased-infected plants under different planting patterns were collected in 2022 and 2023 (Figure 1b). Rhizosphere soil refers to soil that adheres tightly to the root surface. Through both cultivar mixtures, such as, stalk rot-resistant cultivar and a stalk rot-susceptible cultivar, we aimed to explore the mechanisms by which the stalk rot-resistant cultivar enhances disease resistance in the stalk rot-susceptible cultivar. Therefore, two distances from the plant treatment were set up for the bulk soil. Bulk soils were sampled 15 cm and 30 cm away from plants (Figure 1b). Rhizosphere soil and bulk soils were sampled, followed Jia et al. (2024), with six subsamples for bulk soils and three subsamples for rhizosphere soils. The soil samples were thoroughly mixed and divided into separate portions. One portion was stored at −80 °C for extracting microbial DNA, while another was kept at −20 °C for analyzing nitrate nitrogen (NO_3_-N) and ammonium nitrogen (NH_4_-N). The remaining samples were air-dried to measure total nitrogen (TN), total phosphorus (TP), total potassium (TK), available phosphorus (AP), and available potassium (AK) as described by Meng et al. (2019).

### 2.7 DNA extraction, amplicon sequencing, and data processing

The PowerSoil® DNA Isolation Kit (MO BIO Laboratories, Inc., Carlsbad, CA, United States) was used to extract DNA from both rhizosphere and bulk soil samples, followed the protocol provided by the manufacturer. The Illumina MiSeq platform, operated by Majorbio (Shanghai, China), was used to assess microbial diversity in bacterial and fungal communities. This analysis involved sequencing techniques that provide insights into community composition, enabling the identification of microbial taxa and their relative abundances. The amplification of bacterial 16S rRNA genes was conducted using the universal primers 338F (5′-ACTCCTACGGGAGGCAGCAG-3′) and 806R (5′-GGACTACHVGGGTWTCTAAT-3′), while fungal 18S rRNA genes were amplified with the primers SSU0817F (5′-TTAGCATGGAATAATRRAATAGGA-3′) and 1196R (5′-TCTGGACCTGGTGAGTTTCC-3′). Raw sequence data were filtered and merged into single reads to generate clean CCS sequences (Bolyen et al., 2019; Magoč and Salzberg, 2011). Unique reads were identified using UCHIME (v4.2), and operational taxonomic units (OTUs) were grouped based on a 97% similarity threshold using the software USEARCH (v10). Taxonomic assignments, OTU abundance, and alpha diversity indices were determined using the Silva database and QIIME (v1.9.1).

### 2.8 Statistical analyses

A three-way ANOVA was carried out to examine the impacts of genotype, planting pattern, sampling distance, and their interactions on alpha diversity indices. Additionally, a two-way ANOVA was used to analyze the effects of genotype, planting configuration, and their interactions on these diversity indices. Both analyses were conducted using OriginLab Pro 2020b software (OriginLab Corp., Northampton, MA, United States).

Constrained analysis of principal coordinates (CAP) was utilized to evaluate the influence of planting patterns, implemented using the R package *phyloseq*. Statistical significance was determined using the *vegan* package (McMurdie and Holmes, 2013; Oksanen et al., 2015) in R (v4.0.2). Differences in microbial community composition were further analyzed through permutational multivariate analysis of variance (PERMANOVA) based on Bray–Curtis dissimilarities, providing insights into significant variations.

Planting pattern-sensitive OTUs (psOTUs) were detected using indicator species analysis with the *indicspecies* package, complemented by likelihood ratio tests (LRTs) performed through the *edgeR* package, following Hartman et al. (2018). Bipartite networks illustrating significant (*p* < 0.05) associations between OTUs and planting patterns were created with the *igraph* package (Csardi and Nepusz, 2006).

Co-occurrence networks were constructed and visualized using the *igraph* package. Separate networks were developed for bacterial and fungal OTUs based on Spearman’s coefficient (≥0.7, *p* ≤ 0.001). Sample sizes included 24 (bulk soil: 6 replicates × 4 planting patterns) and 12 (rhizosphere soil: 3 replicates × 4 planting patterns). Meta-networks combining bacterial and fungal datasets were also generated. Keystone taxa among psOTUs were identified using Zi-Pi metrics with the *ggClusterNet* package (Li et al., 2021). Additionally, networks linking psOTUs with plant and soil traits were constructed using *igraph* and visualized in Cytoscape (v3.10.1; Shannon et al., 2003).

## 3 Results

### 3.1 Microbial diversity and communities

LC and HC cultivars presented strong habitat filtering of microbial communities with specific sets of microbes (Supplementary File 1, Figure S1). Cultivar mixtures showed no significant influence on *α*-diversity indices, including the Sobs index, Shannon index, Chao1 index, and ACE index, for both bacterial and fungal communities in the rhizosphere and bulk soils (Supplementary File 2).

**Figure 2:**
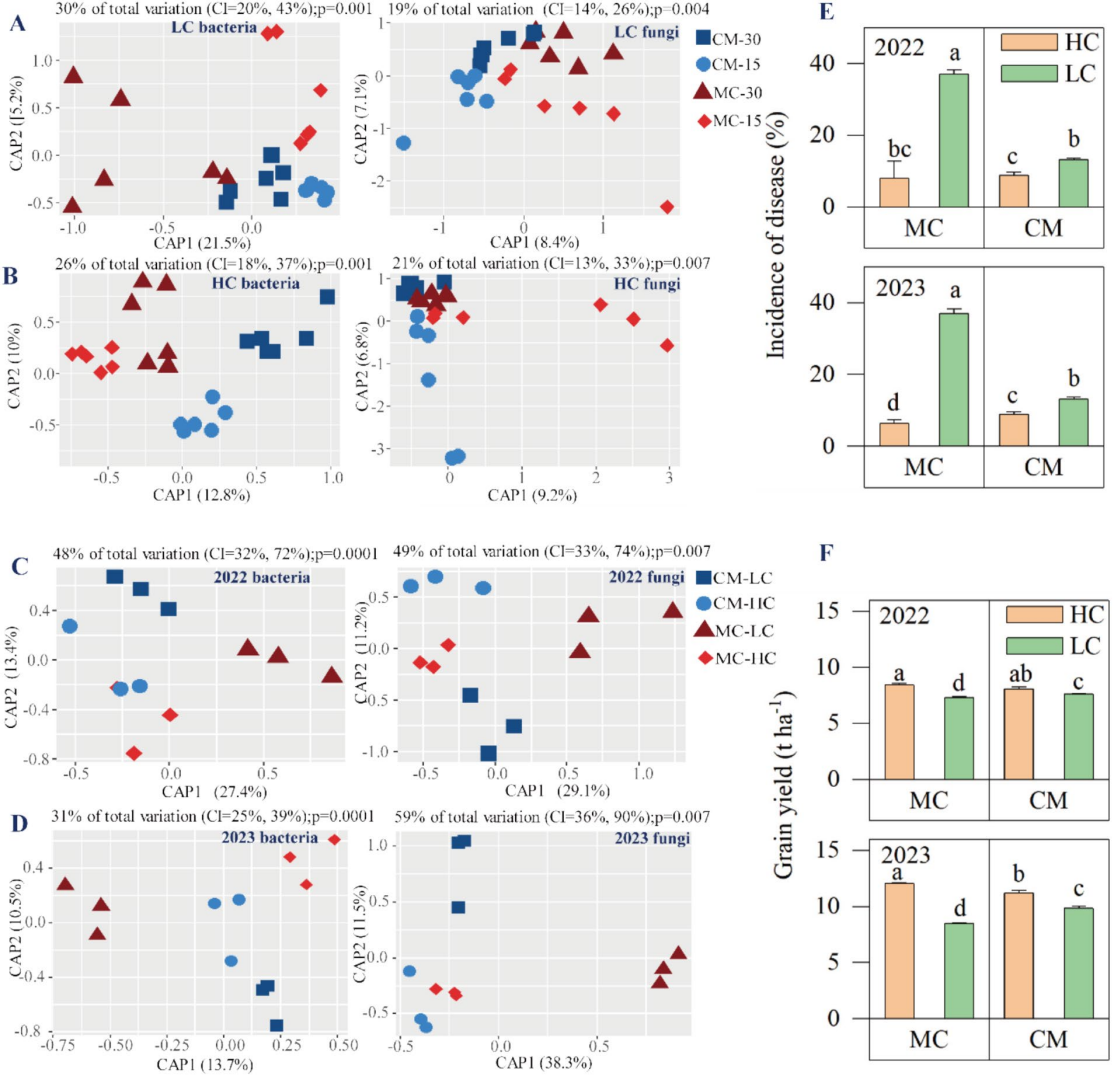
Effects of planting patterns on bacterial and fungal communities of LC and HC in bulk soil [**(A,B)**, *n* = 6], and in the rhizosphere in 2022 and 2023 [**(C,D)**, *n* = 3, incidence of disease **(E)**, and grain yield **(F)**]. MC-HC, DH662 under monocrop; MC-LC, DH701 under monocrop; CM-HC, DH662 under cultivar mixtures; CM-LC, DH701 under cultivar mixtures. MC-15, the soil collected 15 cm away from the roots under monocrop; MC-30, the soil collected 30 cm away from the roots under monocrop; CM-15, the soil collected 15 cm away from the roots under cultivar mixtures; CM-30, the soil collected 30 cm away from the roots under cultivar mixtures.

Furthermore, we analyzed variations in microbial communities by assessing *β*-diversity. CAP using bacteria and fungi OTUs showed that the first two principal coordinates (CAP1 and CAP2) of the total variance in these data of bulk soil or rhizosphere. Different distance groups were observed for the LC and HC microbiota (Figures 2A,B). The distance groups (15 cm and 30 cm) of LC bacteria appeared to be separated by CAP1 (Figure 2A). Response variables to different treatments are separated by CAP1 (Figure 2B). The variation in soil bacterial and fungal communities was significantly influenced by planting patterns (Supplementary File 1, Table S1). CAP analysis distinguished the fungal community based on a distance of 15 cm and 30 cm along axis 2 and samples of 30 cm formed a neighboring cluster between the cultivar mixture and monocrop samples. PERMANOVA further validated the general effect of planting patterns (Supplementary File 1, Table S1). In conclusion, differences influenced by planting patterns and sampling distances were observed in both the LC and HC bacterial and fungal communities, which appeared to primarily influence the bacterial communities.

In 2022, planting patterns significantly accounted for the total variation observed in rhizosphere bacterial and fungal communities, while in 2023, they primarily influenced the fungal community (Supplementary File 1, Table S2). The structure of the bacterial and fungal communities in the LC monocrops was distinctly different from that of cultivar mixtures in both years (Figures 2C,D). For both of bacterial and fungal communities, CAP separated monocrop of LC and cultivar mixtures along axis 1. The effect of planting patterns on fungal communities was greater in 2023 than in 2022. The stalk rot disease incidence of cultivar LC under monocrop was significantly higher than that under cultivar mixtures in 2022 and 2023 (Figure 2E). Conversely, the yield of LC in cultivar mixtures was significantly higher than that in monocrop in 2022 and 2023 (Figure 2F).

### 3.2 Sensitive species

**Figure 3:**
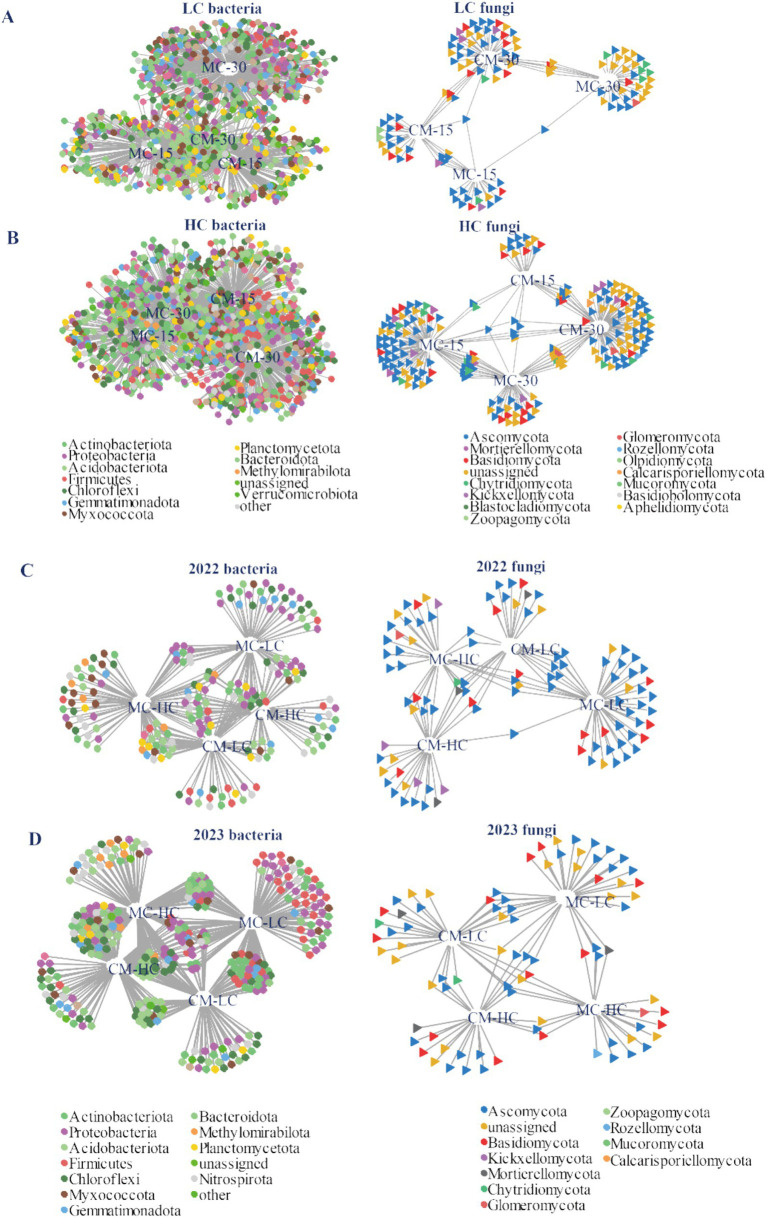
Effects of planting patterns and distance groups on bacterial and fungal bipartite networks of LC and HC in bulk soil [**(A,B)**, *n* = 6], and in the rhizosphere in 2022 and 2023 [**(C,D)**, *n* = 3]. MC-HC, DH662 under monocrop; MC-LC, DH701 under monocrop; CM-HC, DH662 under cultivar mixtures; CM-LC, DH701 under cultivar mixtures. MC-15, the soil collected 15 cm away from the roots under monocrop; MC-30, the soil collected 30 cm away from the roots under monocrop; CM-15, the soil collected 15 cm away from the roots under cultivar mixtures; CM-30, the soil collected 30 cm away from the roots under cultivar mixtures.

Bipartite network presented differences in bacterial and fungal abundances of LC and HC under different planting patterns and distance groups (Figure 3; Supplementary Files 3, 4).

The large number of bacteria common to both the cultivar mixtures and the monocrop at the 15 cm soil position in LC suggests that these samples formed a closely clustered grouping in the ordination (Figure 3A). The lower number of fungal OTUs that were shared between the 15 cm soil position and the 30 cm soil position indicated the scattered distribution of these samples in the ordination (Figures 3A,B). The substantial overlap in rhizosphere bacterial and fungal OTUs between LC under cultivar mixtures and HC under both monocrop and cultivar mixtures suggests that these samples are closely grouped in the ordination (Figures 3C,D). In line with the finding that planting patterns accounted for variation in soil and rhizosphere bacterial communities, a significant number of indicative OTUs were identified as specific to cultivar mixtures in both bulk soil and rhizosphere soil (Supplementary Files 3, 4).

Sensitive species were identified by both methods of indicator OTUs based on indicspecies and edgeR (Supplementary Files 3, 4). In LC, a total of 819 sensitive species were identified for bacteria, while 77 sensitive species were detected for fungi (Supplementary File 3). A total of 967 and 154 sensitive species for bacteria and fungi in HC, respectively, were identified (Supplementary File 3). Consistent with the findings that planting patterns showed different effects on LC and HC communities, sensitive species showed an overlap of 265 bacteria and 13 fungi comparing LC and HC samples. Overall, each planting pattern support a distinct subset of bacterial and fungal communities in the soil and rhizosphere.

### 3.3 Soil and rhizosphere microbial co-occurrence patterns

The individual co-occurrence networks of bacteria and fungi showed that sensitive species of the communities responded very differently in both bulk and rhizosphere soils (Supplementary File 1, Figure S4). Planting patterns changed the bacterial and fungal co-occurrence networks and their properties (Supplementary File 1, Figure S4). The bacterial networks in both LC and HC contained a greater number of significantly co-occurring OTUs compared to the smaller numbers observed in the fungal networks. Similarly, the networks of both cultivars were also more complex in the bacterial community than that in the fungal community in the rhizosphere (Supplementary File 1, Figure S4). Consequently, the bacteria networks of both cultivars were more complex (Supplementary File 1, Figure S4).

**Figure 4:**
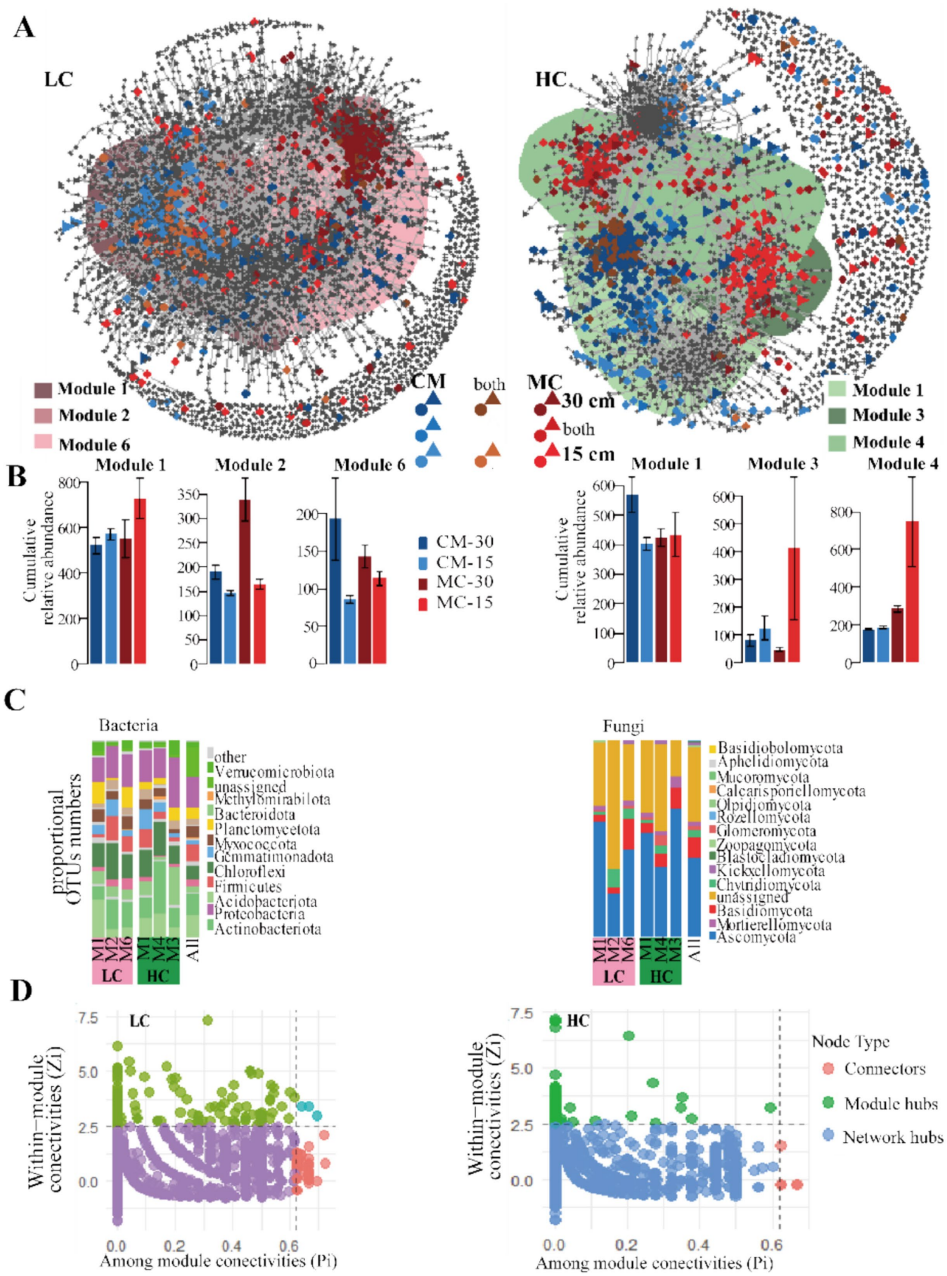
Effects of planting patterns and distance groups on co-occurrence networks in bulk soil [**(A,B)**, *n* = 6] **(A),** cumulative relative abundance in modules **(B),** relative abundance at phylum level of all bacteria and fungi of the planting pattern sensitive modules **(C)**, and Zi–Pi plot of putative keystone OTUs of bacterial and fungal genera in LC and HC networks in bulk soil **(D)**. **(A)** Co-occurrence networks were constructed to display significant correlations (*ρ* > 0.7, *p* < 0.001, represented by gray lines) between maize cultivar mixtures and monocrops. The nodes were color-coded based on treatments. **(B)** Y-axis in ×1,000. HC, DH662; LC, DH701; MC, monocrop; CM, cultivar mixtures; MC-15, the soil collected 15 cm away from the roots under monocrop; MC-30, the soil collected 30 cm away from the roots under monocrop; CM-15, the soil collected 15 cm away from the roots under cultivar mixtures; CM-30, the soil collected 30 cm away from the roots under cultivar mixtures. **(C)** The blue, red, and brown circles or triangles represented the bacterial OTUs or fungal OTUs belong to CM, MC, and both planting patterns.

**Figure 5:**
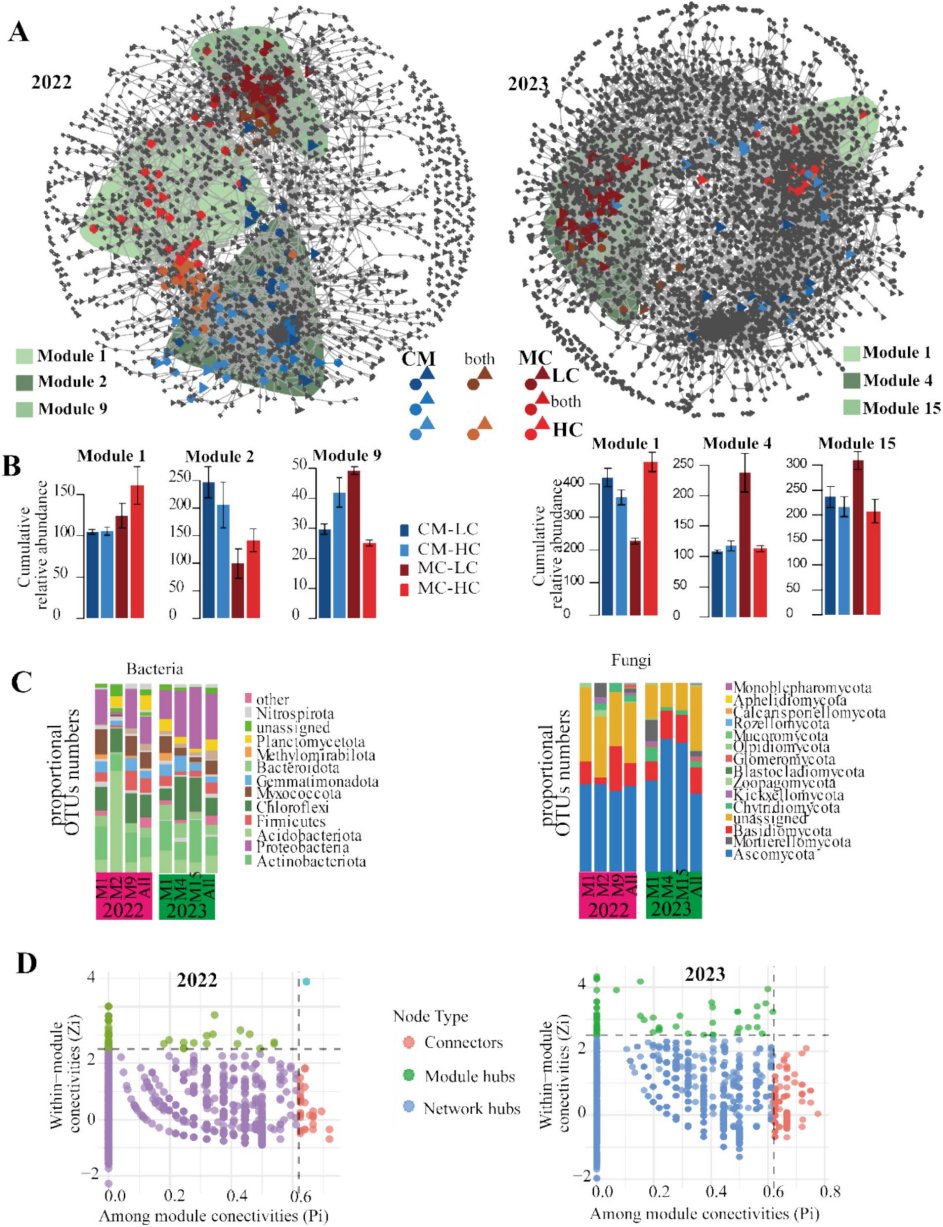
Effects of planting patterns and sampling location groups on co-occurrence network in rhizosphere (*n* = 3) **(A)**, cumulative relative abundance in modules **(B)**, relative abundance at phylum level of all bacteria and fungi in the planting patterns sensitive modules **(C)**, and Zi–Pi plot of putative keystone OTUs of bacterial and fungal genera in LC and HC networks in rhizosphere **(D)**. **(A)** Co-occurrence networks were constructed to display significant correlations (ρ > 0.7, *p* < 0.001, represented by gray lines) between maize cultivar mixtures and monocrops. The nodes were color-coded based on the treatments. **(B)** Y-axis in ×1,000. HC, DH662; LC, DH701; MC, monocrop; CM, cultivar mixtures; MC-HC, DH662 under monocrop; MC-LC, DH701 under monocrop; CM-HC, DH662 under cultivar mixtures; CM-LC, DH701 under cultivar mixtures. **(C)** The blue, red, and brown circles or triangles represented the bacterial OTUs or fungal OTUs belonging to CM, MC, and both planting patterns.

Furthermore, we investigated the distributions and properties of meta co-occurrence networks containing bacteria and fungi in bulk and rhizosphere soils (Figures 4, 5; Table 2). Planting patterns also influenced the abundance of modules of inter-kingdom microbial interactions. Three modules from each network, which included relatively high percentages of sensitive species, were chosen to illustrate the factors driving community differences. Interestingly, even though soil microbial communities determined at 30 cm from stem were similar between LC and HC, different networks were found at 15 cm from stems (Figures 4A,B). Module 1 contained sensitive species specific to MC15, apparently discretized from module 6 that primarily contained sensitive species specific to MC30 and CM30 in LC network (Figures 4A,B). In HC network, module 1 contained numerous sensitive species specific to CM30, separated from modules 3 and 4 containing primarily MC15 specific OTUs (Figures 4A,B). All the sampling distance and planting patterns responsive modules in LC and HC comprised numerous bacterial and fungal taxonomic compositions, while planting patterns do not specifically influence particular microbial lineages within bulk soil (Figure 4C).

**Table 2:** Properties of LC and HC meta co-occurrence networks in bulk soil and rhizosphere.

Soil types	Cultivar/year	Network nodes		Network edges			Connectivity	Keystone		Sensitive species	
		Bacteria	Fungi	Bac-Bac	Fun-Fun	Bac-Fun		Bacteria	Fungi	Bacteria	Fungi
Bulk soil	LC	5,061	688	15,230	294	2081	6.12	55	1	772 (54)	69 (1)
	HC	3,074	493	12,810	296	1,464	8.17	34	1	706 (1)	109 (0)
Rhizosphere	2022	1983	502	4,747	244	1,419	5.16	23	0	175 (2)	42 (0)
	2023	2,824	542	4,720	253	1726	5.49	31	1	37 (3)	64 (0)

The variation in community structure observed in the CAP ordinations caused by monocrop and cultivar mixture is consistent with the co-occurrence networks (Figures 5A,B). Communities in monocrop of LC were different from cultivar mixture of LC and both of cultivar mixture and monocrop of HC (Figures 5A,B). Module 1 contained sensitive species specific to monocrop, apparently discretized from module 2 that primarily contained sensitive species specific to cultivar mixtures in the 2022 network (Figures 5A,B). In the 2023 network, module 1 contained numerous sensitive species specific to all cultivar mixtures and monocrop of HC, separated from module 4 and module 15 containing primarily specific OTUs of monocrop of LC (Figures 5A,B). The half bacterial taxonomic compositions of module 2 in 2022 were comprised of *Acidobacteriota*. The highest fungal taxonomic compositions of all planting pattern sensitive modules in 2022 and 2023 were comprised of *Ascomycota* (Figure 5C). We also inferred 4 co-occurrence networks in the rhizosphere for different planting patterns by WGCNA method. The topological parameters were highly variable in 2 years. The network modularity of monocrop LC was lower than that of cultivar mixture of LC and both of cultivar mixture and monocrop of HC.

We identified the keystone microbes in the co-occurrence network through zi-pi analysis. The sensitive species were identified among zi-pi and module in the network (Figures 4D, 5D; Supplementary Files 3, 4). In rhizosphere soil, sensitive species in cultivar mixtures exhibited higher abundance of *Alphaproteobacteria* and *Actinobacteria* in 2022, *Ascomycota*, *Actinobacteriota* and *Proteobacteria* in 2023 (Supplementary Files 3, 4), indicating that sensitive species were affected by planting patterns and interannual variability.

### 3.4 Relationships between sensitive OTUs and soil and plant properties

**Figure 6:**
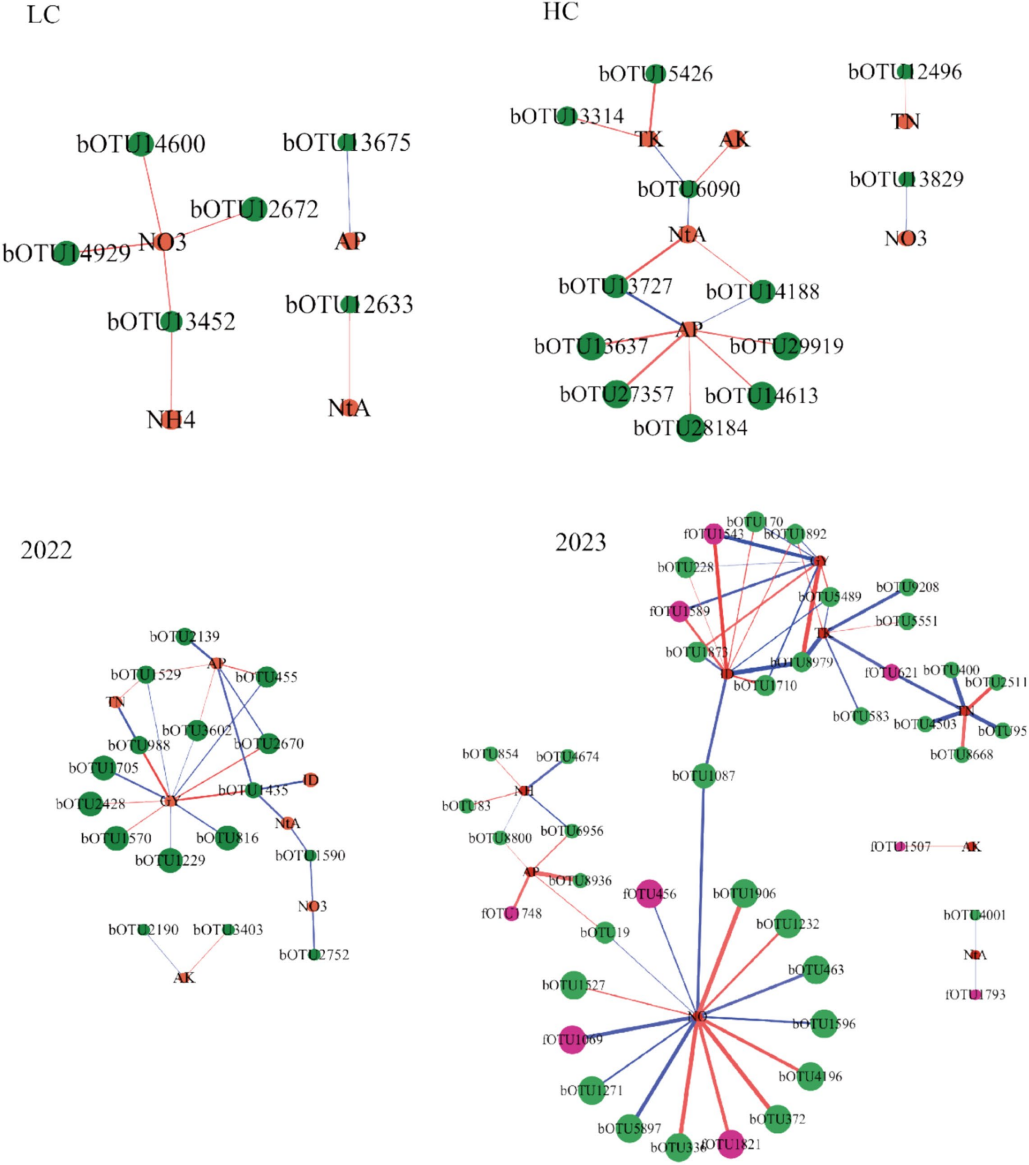
Network analysis reveals the correlation of planting sensitive OTUs with the key properties of soil and plant. The red or blue lines indicate significant positive or negative correlations (ρ > 0.7, *p* < 0.05), respectively. The nodes were the signature OTUs from the microbial network and the key properties of soil and plant. OTUs were colored according to kingdom. Graphics were generated in Cytoscape 3.10.1. The size of each node was degree, and the line width was the weight of the network properties. GY, grain yield; ID, incidence of disease; NH_4_-N, ammonium nitrogen; NO_3_-N, nitrate nitrogen; NA, ratio of nitrate nitrogen to ammonia nitrogen; TN, total nitrogen; TK, total potassium; AK, available potassium; AP, available phosphorus. HC, DH662; LC, DH701; bOTU, bacterial OTU; fOTU, fungal OTU.

The network was constructed to visualize the correlation of sensitive species with the properties of soil and plant, utilizing Spearman’s correlation coefficient (*ρ*) and corresponding *p*-values (Figure 6). Soil nitrate nitrogen, total nitrogen, available phosphorus, grain yield, and incidence of disease possess more complicated relationships with sensitive species in the network in the rhizosphere in 2022 and 2023 (Figure 6). Bacterial OTU1435 (*Adhaeribacter*) and OTU8979 (*Gemmatimonas*) showed a positive association with grain yield and a negative association with disease incidence in the rhizosphere, while bacteria OTU1435 was negatively correlated with available phosphorus and bacteria OTU8979 was negatively correlated with total potassium (Figure 6; Supplementary Files 3, 4). Additionally, the incidence of disease was negatively correlated with fungi OTU1589 (*Fusarium*) and OTU1543 (*Hypocreales*).

## 4 Discussion

### 4.1 Responses of bulk and rhizosphere soil microbiomes to monocrop and cultivar mixtures

The rhizosphere microbiome has played a critical role in plant growth and provided promising solutions to sustainable agriculture (Xun et al., 2024). Regarding the influence of agricultural farming practices on soil microbiome communities, previous studies have primarily focused on the impacts of intercropping between different species on microorganisms in the rhizosphere or bulk soil (Li et al., 2018), root-related microbiota structure, assembly, and variation (Edwards et al., 2014; Peiffer et al., 2013; Walters et al., 2018), intraspecies mixture focused on the variations in either the bacterial or fungal microbiome communities (Jia et al., 2024). This study aims to examine the effects of cultivar mixtures on microorganisms at different sampling distances from susceptible corns, including rhizosphere microorganisms, as well as those in bulk soil at 15 cm and 30 cm. Our findings revealed that microbial diversity in bulk soil varied depending on the distance from the roots of the disease-susceptible cultivar (Figure 2A). Our findings showed significant differences in both fungi and bacteria at 15 cm between cultivar mixtures and monocrops, while only bacteria showed significant differences at 30 cm sampling location. This result indicated that the bacteria in the soil are more susceptible to the impact of maize cultivar mixture, while the fungi are more influenced when they are closer to the roots. As the distance to the root system decreases, microorganism diversity becomes more significant. This may be because more palmitic acid is derived from healthy plant root exudates, which in turn attracted more nitrogen-fixing bacteria (such as *Paenarthrobacter* and *Sphingomonas*) and these bacteria potentially alleviated nitrogen deficiency in the soil, improved crop growth, and enhanced crop defense capabilities (Tong et al., 2024). Consequently, a decline in microbial species diversity had a profound impact on plant health, leading to greater severity and prevalence of diseases caused by soil-borne pathogens (Wei et al., 2019; Zhou et al., 2021). In this research, there were significant differences in the rhizosphere microbial changes between resistant and susceptible varieties. There was no obvious distinction between bacteria and fungi in resistant varieties between different planting patterns, while there were clear distinctions in both bacteria and fungi in the rhizosphere of susceptible varieties. This suggests that the microbial community of susceptible varieties is influenced by resistant varieties. It supported that specialist pathogen dilution generates more productivity (Wang et al., 2023). This indirectly suggested that maize may mediate the changes in soil microorganisms through some root exudates or volatiles from the rhizome (Li et al., 2018; Tong et al., 2024).

### 4.2 Effects of cultivar mixtures on microbial co-occurrence and sensitive microbes compared with monocrops

Plant disease outbreaks had resulted in the loss of primary productivity and biodiversity (Singh et al., 2023). Through co-occurrence network analysis (Hartman et al., 2018; Liu et al., 2023), we identified potential relationships within the microbiome community. Topological parameters of microbial networks can serve as valuable tools for evaluating the complexity and stability of rhizosphere microbial interactions, thereby reflecting the assembly of microbial communities (Qiao et al., 2024). Our study presented that the total number of links (network connectivity) of the bulk soil in disease-resistant varieties was significantly higher than in disease-susceptible varieties, demonstrating that the bulk soil networks of disease-resistant varieties were more complex. While between the two varieties, the total number of links (network connectivity) in the bulk soil was higher than in the rhizosphere soil, indicating that specific recruitment occurred in the rhizosphere microbiota. Furthermore, the network parameters of higher modularity and lower density validated that the rhizosphere networks were more stable under a cultivar mixture of disease-susceptible variety. Co-occurrence networks have proven to be an effective method for investigating abundance patterns in complex microbial communities and offer valuable guidance for planning future experimental designs (Hartman et al., 2018). Consequently, under disease and healthy conditions, cultivar mixtures of maize could recruit various bacterial communities. Our previous research revealed that cultivar mixtures had improved the abundance of *Chitinophagaceae*, creating a relatively healthy rhizosphere bacterial community (Jia et al., 2024). Keystone species are recognized for their extensive interactions with numerous other species, making them integral to the overall stability and function of the community (Berry and Widder, 2014; Ma et al., 2016). In the bulk soil collected 15 cm away from the roots, the cumulative abundance of microbes of the two modules of the disease-susceptible variety decreased, while there was no significant difference in the disease-resistant variety. This suggested that the disease-susceptible microbial co-expression pattern in the bulk soil close to disease-resistant plants was strongly impacted. It was very crucial to understand the reason for the variation.

The rhizosphere networks indicated that the specific modules of disease-susceptible variety were clearly distinguished between cultivar mixtures and monocrop, and the rhizosphere co-occurrence network pattern was closer to resistant variety in the case of cultivar mixtures. This suggests that there has been a significant change in the disease-susceptible rhizosphere microbial recruitment, which was induced by the disease-resistant variety. Simultaneously, disease-susceptible variety had increased the accumulated relative abundance in module 1 in 2022 and 2023 in the case of cultivar mixtures. To further determine network keystone taxa, assemble, network zipi analysis and edgeR analysis were performed. The results showed that cultivar mixtures had significantly increased the relative abundance of advantageous microorganism, such as *Chitinophagaceae*, *Gemmatimonas*, *Adhaeribactor,* and *Microscillaceae*. Microorganisms *Chitinophagaceae* have been shown to consistently suppress fungal diseases (Carrión et al., 2019). The *Microscillaceae* had the relatively lower abundances in the parasitized samples compared with the non-parasitized samples (Xi et al., 2022). Family *Microscillaceae* belongs to order *Cytophagales* and phylum *Bacteroidetes* and Family *Microscillaceae* was the biomarker (Li et al., 2023). *Microscillaceae* was likely related to the increase of alkaline phosphatase activities (Wang et al., 2024). The *pufM* gene, which codes for the M subunit of the bacterial type-II reaction center, serves as a widely used molecular marker for identifying phototrophic *Proteobacteria*, *Chloroflexota*, and *Gemmatimonas* (Mujakić et al., 2022). A more widely applicable marker is the gene *bchY*, which encodes the *Y* subunit of chlorophyllide reductase, as it targets all anoxygenic phototrophic organisms (Yutin et al., 2009). The microbes with the ability to degrade autotoxins had been shown to exhibit beneficial effects on crop growth and soil health, such as *Pseudomonas*, *Trichoderma,* and *Adhaeribacter*, which can degrade phenolic acids, biodegradation potential (Liu et al., 2017; Xuan et al., 2024; Zhu et al., 2023). *Adhaeribacter* has been previously linked to increased urease and alkaline phosphomonoesterase activities (Rodríguez-Caballero et al., 2017; Sun et al., 2014), which play a role in maintaining nitrogen and phosphorus levels in the soil (Lin et al., 2021). These characteristics make *Microscillaceae*, *Adhaeribacter, and Chitinophagaceae* excellent agent for promoting crop growth development by regulating the nutrients structure in rhizosphere soil and suppressing the incidence of stalk rot. Our results revealed that cultivar mixtures could induce the recruitment of the rhizosphere keystone taxa. These core species enhance the resistance of disease-susceptible varieties to pathogenic bacterium, via the unique inductive effect in the case of cultivar mixtures. Focusing research and practical applications on keystone taxa can be considered an effective strategy for promoting healthy crop development.

## 5 Conclusion

Cultivar mixtures altered bacterial and fungal community structure with the monocrop of the susceptible variety indicating the facilitating effect of within-species diversification. The keystone taxa identified by complementary approaches were associated with the changing physicochemical status in the rhizosphere. The enriched taxa of *Gemmatimonas* and *Adhaeribacter* were negatively related to the incidence of maize stalk rot disease. Our findings demonstrated that cultivar mixtures in maize harbored more plant growth promoting microbes that improve plant disease resistance. Future research should aim to identify the dominant microbes that can be manipulated through cultivar mixtures in order to enable widespread agricultural sustainability.

## Data Availability

The original contributions presented in the study are publicly available. This data can be found here: https://www.ncbi.nlm.nih.gov/, Accession number PRJNA1364799.
