# Supplementary material for: Suppressing maize stalk rot through promoted rhizosphere microbial recruitment in cultivar mixtures
Source: Front Microbiol. 2025 Dec 2;16:1627153. doi: 10.3389/fmicb.2025.1627153 (PMC12705605; doi:10.3389/fmicb.2025.1627153)
Supplement: Supplementary file 4 [file Data_Sheet_1.docx]

**
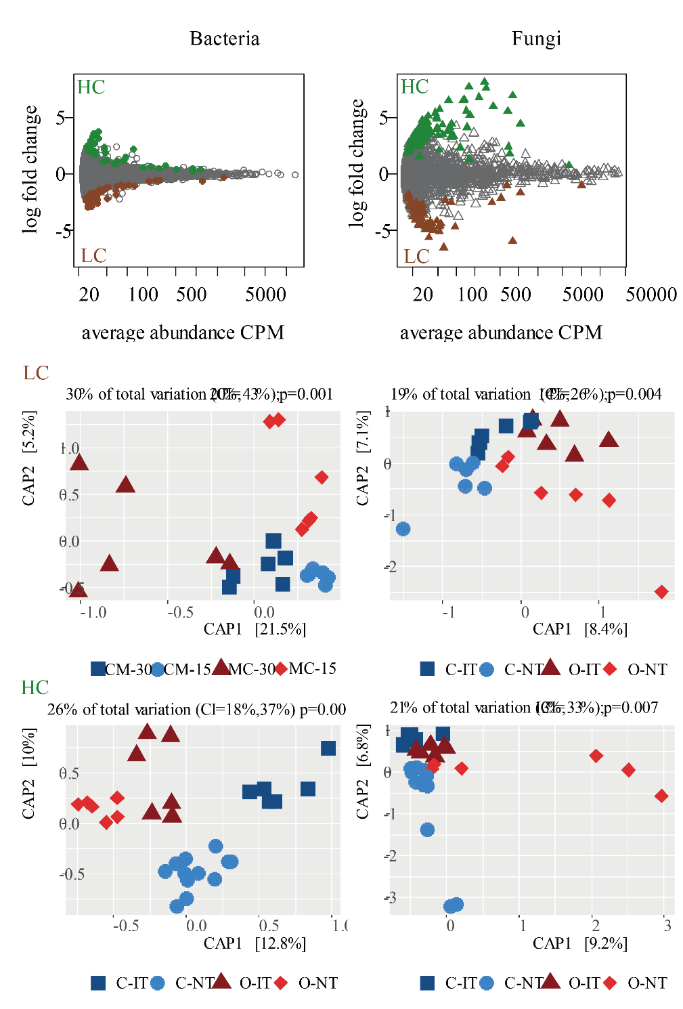
**Figure S1 LC and HC cultivar harbor specific sets of microbes in bulk soil. MA plots displaying the abundance patterns of bacteria and fungi in bulk soil microbiomes in LC and HC cultivar. X-axis reports average OTU abundance (as counts per million, CPM), and Y-axis log2-fold change (HC relative to LC). HC and LC-specific OTUs were colored in green and brown, respectively, and non-differentially abundant OTUs are in gray (likelihood ratio test, p < 0.05, FDR corrected)


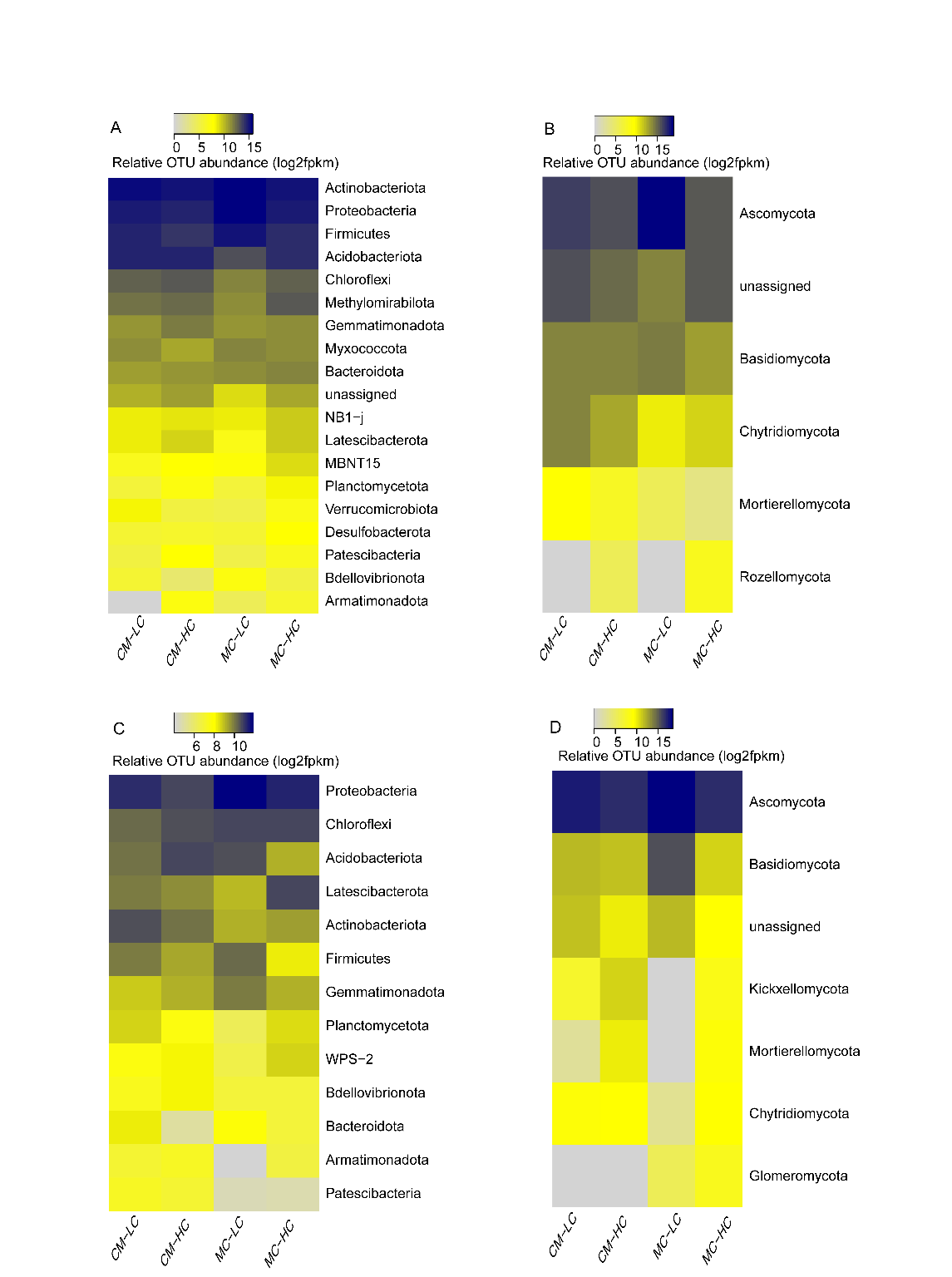


Figure S2 The microbial composition in rhizosphere in 2022 (A and B) and 2023 (C and D).


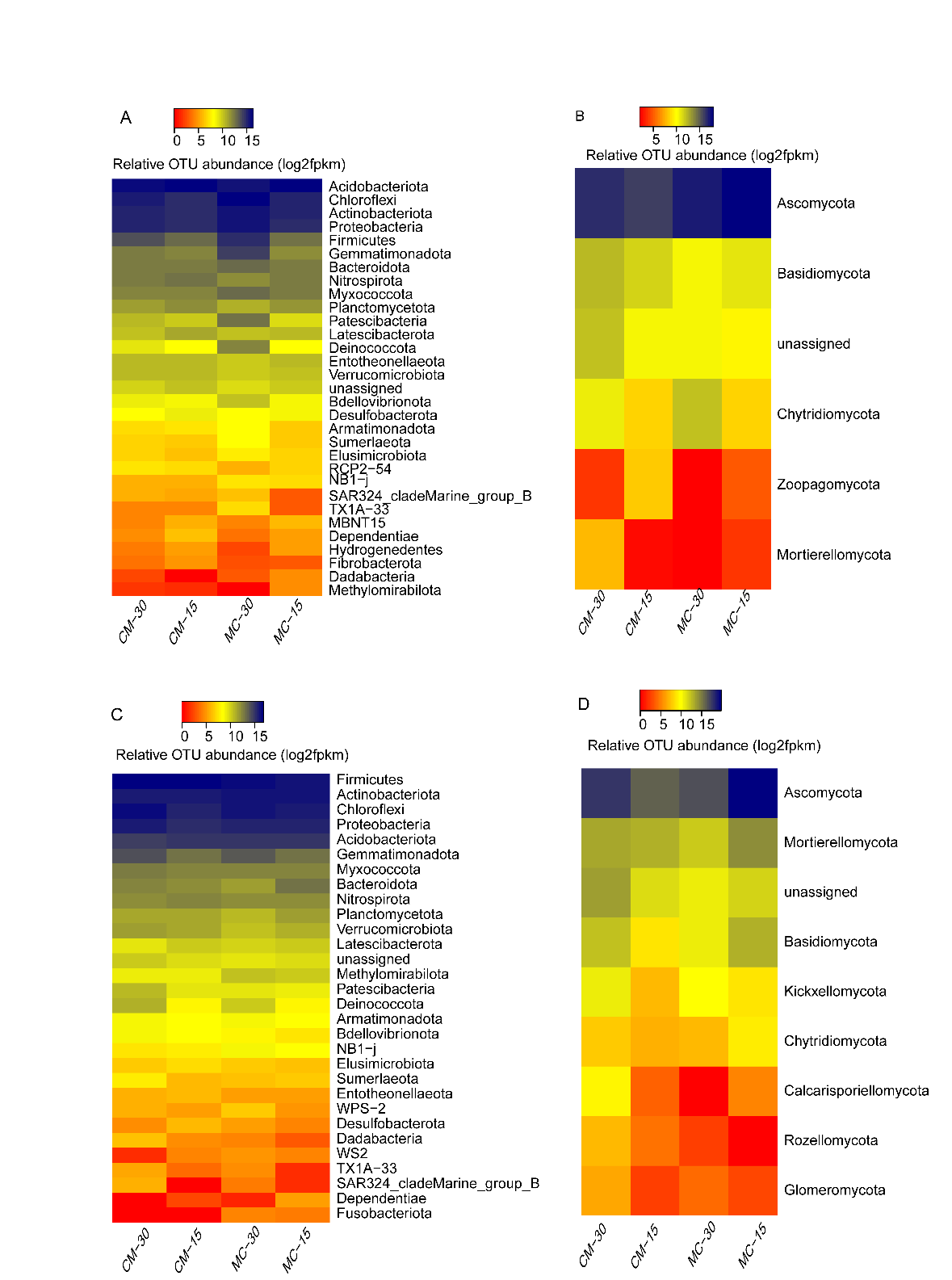
Figure S3 The microbial composition of LC (A and B) and HC (C and D) cultivar in bulk soil..


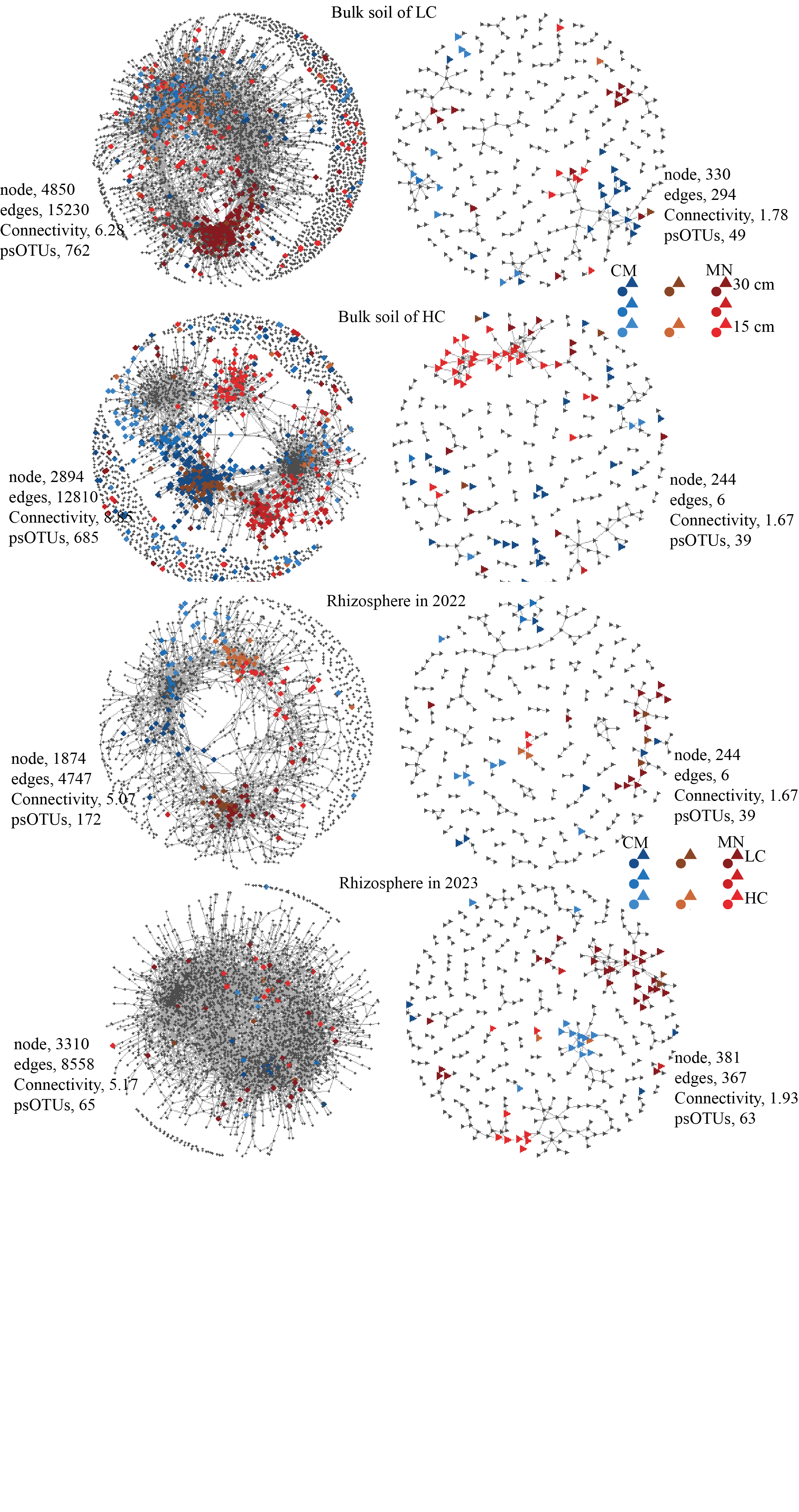
Figure S4 Co-occurrence networks visualizing significant correlations (ρ>0.7, p<0.001; indicated with grey lines) between OTU pairs in the soil and rhizosphere bacterial and fungal communities. Circles and triangles represent bacteria and fungi OTUs, respectively. OTUs were colored by their association to the different planting patterns and genotypes.

Table S1 Results of PERMANOVA testing the effects of Planting patterns and Distance on bacterial and fungal communities in soil and rhizosphere samples. Significant effects are indicated in bold (*p<0.05, **p<0.01, ***p<0.001). Different letters in the pairwise comparisons indicate significant differences at p<0.05 (FDR corrected). Results of BETADISP testing for differences in multivariate dispersion between cropping systems in root and soil samples in bacterial and fungal communities.

|  | LC | | | | HC | | | |
| --- | --- | --- | --- | --- | --- | --- | --- | --- |
|  | Bacteria  pseudo-F | R^2^ | Fungi  pseudo-F | R^2^ | Bacteria  pseudo-F | R^2^ | Fungi  pseudo-F | R^2^ |
| Planting patterns (P) | 2.66** | 0.09 | 1.95* | 0.08 | 4.10*** | 0.12 | 2.31* | 0.07 |
| Distance (D) | 4.11*** | 0.14 | 1.77* | 0.07 | 3.64*** | 0.10 | 2.45* | 0.07 |

Table S2 Results of PERMANOVA testing the effects of Planting patterns and Genotypes on bacterial and fungal communities in soil and rhizosphere samples. Significant effects are indicated in bold (*p<0.05, **p<0.01, ***p<0.001). Different letters in the pairwise comparisons indicate significant differences at p<0.05 (FDR corrected). Results of BETADISP testing for differences in multivariate dispersion between cropping systems in root and soil samples in bacterial and fungal communities.

|  | 2022 | | | | 2023 | | | |
| --- | --- | --- | --- | --- | --- | --- | --- | --- |
|  | Bacteria  pseudo-F | R^2^ | Fungi  pseudo-F | R^2^ | Bacteria  pseudo-F | R^2^ | Fungi  pseudo-F | R^2^ |
| Planting patterns (P) | 2.64** | 0.18 | 1.65 | 0.12 | 1.07 | 0.09 | 3.20* | 0.20 |
| Genotype (G) | 2.73** | 0.19 | 3.07** | 0.22 | 1.16 | 0.10 | 3.67** | 0.23 |

Table S3 Soil and plant parameters in 2022

|  | NH4 | NO3 | NA | TN | AP | AK | TK | ID | GY |
| --- | --- | --- | --- | --- | --- | --- | --- | --- | --- |
| MC-HC | 8.48 | 13.6 | 1.6 | 0.89 | 17.22 | 195.84 | 10.05 | 4.46 | 12.06 |
| MC-LC | 7.80 | 24.47 | 3.14 | 1.16 | 24 | 256.93 | 10.32 | 30.64 | 8.51 |
| CM-HC | 8.34 | 23.66 | 2.84 | 1.08 | 17.27 | 155.06 | 10.05 | 8.08 | 11.18 |
| CM-LC | 7.44 | 18.18 | 2.45 | 1.35 | 17.58 | 154.85 | 10.59 | 7.94 | 9.83 |

Note:GY, grain yield; ID, incidence of disease; NH4, ammonia nitrogen; NO3, nitrate nitrogen; NtA, ratio of nitrate nitrogen to ammonia nitrogen TN, total nitrogen; TK, total potassium; AK, available potassium; AP, available phosphorus.

Table S4 Soil and plant parameters in 2023

|  | NH4 | NO3 | NA | TN | AP | AK | TK | ID | GY |
| --- | --- | --- | --- | --- | --- | --- | --- | --- | --- |
| MC-HC | 6.81 | 14.43 | 2.12 | 1.07 | 20.63 | 236.22 | 10.91 | 6.43 | 8.43 |
| MC-LC | 12.25 | 20.15 | 1.64 | 1.07 | 18.99 | 420.25 | 11.54 | 37.06 | 7.41 |
| CM-HC | 12.49 | 21.26 | 1.7 | 1.01 | 17.32 | 215.70 | 10.94 | 8.87 | 8.05 |
| CM-LC | 6.85 | 16.63 | 2.43 | 1.06 | 23.98 | 215.44 | 10.89 | 13.18 | 7.60 |

Note:GY, grain yield; ID, incidence of disease; NH4, ammonia nitrogen; NO3, nitrate nitrogen; NtA, ratio of nitrate nitrogen to ammonia nitrogen TN, total nitrogen; TK, total potassium; AK, available potassium; AP, available phosphorus.
